# Inversed impaired osteogenic activity in children with severe obesity due to *MC4R* deficiency compared to *LEP* and *LEPR* deficiency

**DOI:** 10.1038/s41366-026-02047-w

**Published:** 2026-03-13

**Authors:** Qasim M. Janjua, Roohia Khanam, Sadia Saeed, Jaida Manzoor, Attiya Haseeb, Sharoon Hanook, Amelie Bonnefond, Muhammad Arslan, Philippe Froguel

**Affiliations:** 1 Department of Physiology and Biophysics, College of Medicine and Health Sciences, National University of Science and Technology, Sohar, Oman; 2 KAM School of Life Sciences, Forman Christian College, Lahore, Pakistan; 3 Inserm UMR 1283, CNRS UMR 8199, EGID, Institut Pasteur de Lille, Lille, France; 4 University of Lille, Lille University Hospital, Lille, France; 5 Department of Metabolism, Digestion and Reproduction, Imperial College London, London, UK; 6 Department of Paediatric Endocrinology, Children’s Hospital, Lahore, Pakistan; 7 Department of Statistics, Forman Christian College, Lahore, Pakistan

**Keywords:** Obesity, Paediatrics, Obesity

## Abstract

**Objective:**

Chronic obesity is associated with impaired bone health. However, few investigations have been conducted to assess bone physiology in early-onset obesity. In this study, we measured specific bone turnover and metabolic biomarkers in children with severe obesity with biallelic loss-of-function variants of the leptin (*LEP)*, leptin receptor *(LEPR)*, or melanocortin 4 receptor (*MC4R)* genes.

**Methods:**

Thirty-nine children aged 0.3–8.8 years with a BMI SDS ≥ 3, previously identified with pathogenic variants in *LEP*, *LEPR*, or *MC4R*, were recruited for the current study. Additionally, 13 age-matched children with severe obesity who tested negative for variants in known obesity-related genes were included, and another 13 unrelated age-matched children with normal body weight served as the control group. Serum osteocalcin, osteopontin, osteoprotegerin, and sclerostin levels were assessed using multi-analyte profiling. Serum leptin, insulin, and cortisol levels were determined using ELISA.

**Results:**

Serum levels of osteocalcin and osteopontin, specific markers of bone formation, were significantly lower in children with *LEP* and *LEPR* biallelic variants than in the control group. In contrast, the values of these two biomarkers in children with *MC4R* deficiency were significantly higher than those in the other groups. No differences were observed in the bone resorption markers osteoprotegerin and sclerostin. Hyperleptinemia was more pronounced in children with *LEPR* deficiency. Serum insulin concentrations were elevated in individuals with *MC4R* deficiency, whereas serum cortisol levels were significantly higher in children with *LEP* deficiency than in all other groups.

**Conclusion:**

Our data demonstrate that osteogenic activity (but not resorption activity) is differentially affected in children with complete genetic disruption of the leptin-signaling pathway. Children with *MC4R* deficiency showed higher osteogenic markers, but children with *LEP* and *LEPR* deficiencies showed the opposite. Our results support the usefulness of bone turnover biomarkers for the assessment and management of bone health in different types of obesity. *Keywords:* Monogenic obesity, Bone metabolism biomarkers, *LEP, LEPR, MC4R*

## Introduction

The prevalence of childhood and adolescent obesity has escalated significantly in the wake of the obesity pandemic over the past three decades and is emerging as a major global public health concern. According to the World Health Organization, by 2022, 37 million children with obesity were under 5 years of age, and more than 390 million young children and adolescents (5–19 years) were classified as overweight [1]. Currently, the global increase in the prevalence of childhood obesity is anticipated to reach ~380 million by 2035 [2]. Childhood obesity substantially increases the risk of chronic diseases in young or middle-aged adults, such as type 2 diabetes, cardiovascular diseases, certain forms of cancer, sleep apnea, psychological distress, and other metabolic dysfunctions [3]. Notably, children with obesity are more susceptible to health-related complications than adults [4]. In this regard, we recently demonstrated that childhood obesity caused by recessive genetic defects may be associated with dramatic mortality and chronic and infectious disease rates, largely due to lung and gut infections [5, 6].

Previous studies also suggested that obesity influences bone metabolism and modulation, potentially leading to bone abnormalities [7]. Historically, obesity has been thought to have an advantageous and positive role in sustaining bone health by averting bone loss and osteoporosis due to mechanical load [8, 9]; however, several recent studies have indicated that surplus fat mass is related to low bone mineral density and total mineral content [10, 11]. The dynamic process of bone remodeling, essential for maintaining bone strength, involves the coordinated activities of osteoblasts forming new bone and osteoclasts resorbing old bone [12]. This process is regulated by the interplay between neural, endocrine, and paracrine signals, thus emphasizing the complex relationship between body composition and skeletal integrity.

Among other factors, the adipocyte-derived hormone leptin (encoded by *LEP*) plays an important role in bone metabolism. The involvement of leptin signaling in bone metabolism is further strengthened by the presence of the leptin receptor (LEPR) [13] in ossifying cartilage [14], chondrocytes, and adult primary osteoblasts [15]. Previous studies have demonstrated that leptin influences the dynamics of bone remodeling, thus affecting both bone formation and resorption [16–18]. Peripheral and central administration of leptin has been shown to increase osteogenesis, bone density, and mineralization in mice with *Lep* deficiency (ob/ob) [19–21]. The effects of leptin replacement on bone metabolism in patients with *LEP* deficiency have been sparse, mostly inconsistent, and related to case reports or small series of patients from diverse ethnicities (or origins) [22–24].

Contrary to individuals with *LEP* deficiency, children with melanocortin 4 receptor (MC4R) deficiency have high bone mass linked to a decrease in bone resorption, attributed mainly to overexpression of the neuropeptide cocaine- and amphetamine-regulated transcript (CART) in the hypothalamus [25, 26]. *Mc4r*^−/−^ mice also have increased hypothalamic CART and exhibit high bone mass due to the inhibition of osteoclast proliferation and function [25]. The bone phenotype of mice with *Mc4r* deficiency has been shown to be corrected by decreasing CART expression [26], demonstrating that increased bone mass due to *Mc4r* deficiency is mainly mediated by increased CART signaling.

Most studies on bone metabolism in the context of obesity have utilized dual-energy X-ray absorptiometry to assess bone mass and mineral density, but lack insight into the physiological processes of bone resorption and formation [27–29]. Only a few studies have been conducted on bone health in children with early-onset severe obesity owing to monogenic or undetermined plausible genetic causality [30, 31]. However, the available data are conflicting and based on case reports or a small sample size [22, 32, 33]. Furthermore, a limited number of studies have addressed changes in bone metabolism related to increased fat mass using bone turnover markers in children with severe forms of obesity [34, 35]. Bone markers such as osteocalcin (OC), bone-specific alkaline phosphatase, and osteopontin (OPN) [36–38] have been used to assess bone formation, whereas sclerostin (SOST) and osteoprotegerin (OPG) are considered biomarkers for bone resorption [39, 40].

Furthermore, there is a paucity of information regarding the dynamics of bone turnover in children with monogenic forms of obesity [4, 41]. Errors in bone metabolism in children at an early age can have long-term consequences for bone health, potentially increasing the risk of fractures and osteoporosis later in life [7]. Here, we assessed bone health using bone turnover markers in an ethnically homogenous cohort of children from consanguineous families from Pakistan carrying pathogenic biallelic variants in *LEP*, *LEPR*, or *MC4R*, drawn from one of the world’s largest cohorts of children with severe obesity [5]. Additionally, we have included age-matched children with severe obesity who were negative for variants in these three genes or other known genetic variants associated with obesity and a group of children with normal body weight as controls. All study participants shared a common ethnicity and similar socio-economic and environmental conditions.

## Materials and methods

### Participants

This study included 52 children from the Severe Obesity in Pakistani Population (SOPP) cohort belonging to consanguineous families with a BMI standard deviation score (SDS) ≥ 3 and a history of hyperphagia. Among these, 39 children were previously identified to have homozygous loss-of-function variants in the *LEP*, *LEPR*, or *MC4R* genes (LEP: NM_000230.3; LEPR: NM_002303.6; MC4R: NM_005912.3) [5]. In addition, 13 age-matched children with severe obesity who tested negative for variants in these three genes or other known monogenic causes of severe obesity based on whole-exome sequencing were included. Thirteen age-matched children with normal body weight from the same population served as the control group. Patients and/or their parents were interviewed regarding their family and medical history. Physical examination and anthropomorphic measurements were performed. A 4–5 ml blood sample was obtained between 1000 and 1200 h, in each case, for serum extraction and subsequent biochemical estimations.

### Biochemical determinations

Simultaneous quantification of the bone turnover markers OC, OPN, OPG, and SOST was carried out in serum samples by multi-analyte profiling using EMD Millipore’s MILLIPLEX MAP Human Bone Magnetic Bead Kit (Cat. # HBNMAG-51K) (Billerica, MA, USA). Luminex analyzer MAGPIX (Austin, Texas, USA) was used for reading the plate. Serum levels of leptin, insulin, and cortisol were measured by enzyme-linked immunosorbent assay (ELISA) using commercially available kits (leptin, Labor Diagnostika Nord GmbH, Nordhorn, Germany; insulin and cortisol, Monobind Inc., Lake Forest, CA, USA) and an automated EIA analyzer (Bio-Rad Laboratories, Hercules, CA, USA). All assays were performed in duplicate according to the manufacturer’s instructions. The inter- and intra-assay variations were <11% in all cases.

### Statistical analysis

The significance of the differences between the groups was analyzed using Scheffe’s multiple comparison test. Statistical significance was set at *P* < 0.05. All calculations were performed using Statistical Package for the Social Sciences version 20 (SPSS, Inc., Chicago, IL, USA).

### Ethical approval

The study protocol was approved by the Institutional Research Board (IRB): Forman Christian College (A Chartered University), Lahore, Pakistan (Ref: 817/0325), Ethical Research Committee (ERC): KAM School of Life Sciences, Forman Christian College (A Chartered University), Lahore, Pakistan (Ref: 60/0721) and Ethical Committee, Children’s Hospital and Institute of Child Health, Lahore, Pakistan (Ref: 5178). Written informed consent to participate in this study was obtained from all patients/parents. The study was performed in accordance with the principles of the Declaration of Helsinki.

## Results

### Physical characteristics

The physical characteristics of children with monogenic obesity caused by *LEP*, *LEPR*, or *MC4R* biallelic variants, age-matched individuals with severe obesity with unknown genetic etiology, and those with normal body weight are summarized in Table 1. Although all affected individuals were characterized by early-onset severe obesity, the majority of children with *LEP* and *LEPR* deficiencies presented with hyperphagia at a much earlier stage than children with *MC4R* deficiency [5] (Table 1). Notably, there was an increase in linear growth in children with *MC4R* deficiency but not with *LEP* or *LEPR* deficiency, compared to age-matched (4.3–7.8 years) children with normal body height (124.9 ± 4.4 vs 109.0 ± 1.6 cm).

**Table 1:** Physical characteristics of children with severe obesity.

	Subjects with variants in				
Characteristic	*LEP*	*LEPR*	*MC4R*	SvOb	NWC (wild type)
*N*	21	10	8	13	13
M/F ratio	9/12	6/4	4/4	8/5	7/6
Age range (years)	0.3–4.4	0.7–5.1	3.9–8.8	0.7–8.0	4.3–7.8
Age (years)	1.2 ± 0.2^c, d, e^	1.8 ± 0.4^c, e^	6.7 ± 0.6^a, b, d^	3.3 ± 0.7^a, c, e^	6.0 ± 0.3^a, b, d^
Height (cm)	76.8 ± 2.6^c, d, e^	83.3 ± 5.8^c, e^	124.9 ± 4.4^a, b, d^	96.0 ± 6.2^a, c^	109.0 ± 1.6^a, b^
Weight (kg)	19.4 ± 2.1^c^	20.7 ± 2.7^c^	57.1 ± 7.2^a, b, d, e^	31.0 ± 4.3^c^	19.4 ± 1.2^c^
BMI SDS^f^ for age	8.1 ± 0.6^e^	7.5 ± 1.0^e^	7.8 ± 0.7^e^	8.1 ± 0.9^e^	0.4 ± 0.3^a, b, c, d^
BMI	31.4 ± 1.3^e^	29.5 ± 1.7^e^	35.5 ± 2.2^e^	31.4 ± 1.6^e^	16.2 ± 0.7^a, b, c, d^

Significant difference (Scheffe’s multiple *t* test; *P* < 0.05): ^a^ *vs LEP*; ^b^ *vs LEPR*; ^c^ *vs MC4R;* ^d^ *vs* SvOb, ^e^ *vs* NWC.

^f^WHO Anthro, v 3.2.2; AnthroPlus, v 1.0.4; Values represent mean ± SEM.

### Bone turnover biomarkers

Mean serum OC concentrations in individuals with *MC4R* deficiency were >3-fold higher compared to children with *LEP* or *LEPR* deficiency and were also significantly raised over the individuals with severe obesity negative for these variants and children with normal body weight (185.2 ± 25.3 vs 70.4 ± 7.2 and 106.7 ± 11.5 ng/ml, respectively). Remarkably, the levels of this biomarker of bone formation were significantly lower in children with *LEP* and *LEPR* variants than in children with normal body weight (Table 2). The mean serum OC levels in children with severe obesity with unknown genetic etiology were intermediate to the control values and in those with *LEP* or *LEPR* deficiency.

**Table 2:** Metabolic hormone levels in children with severe obesity.

	Subjects with variants in				
Characteristic	*LEP*	*LEPR*	*MC4R*	SvOb	NWC (Wild type)
*N*	21	10	8	13	13
Osteocalcin (ng/ml)	54.1 ± 7.5^c, e^	43.1 ± 8.5^c, e^	185.2 ± 25.3^a, b, d, e^	70.4 ± 7.2^c^	106.7 ± 11.5^a, b, c^
Osteopontin (ng/ml)	7.9 ± 0.8^c, e^	6.8 ± 0.9^c, e^	18.4 ± 0.7^a, b, d^	8.0 ± 0.7^c, e^	17.7 ± 2.3^a, b, d^
Sclerostin (pg/ml)	1048 ± 99	1164 ± 142	713 ± 61	1176 ± 132	952 ± 147
Osteoprotegerin (pg/ml)	371 ± 13	371 ± 17	387 ± 25	390 ± 14	408 ± 30
Leptin (ng/ml)	0.0 ± 0.0^b, c, d^	50.5 ± 5.4^a, c, d, e^	32.5 ± 5.6^a, b, e^	20.5 ± 3.1^a, b, e^	2.7 ± 0.3^b, c, d^
Insulin (uIU/ml)	13.0 ± 2.3^c^	17.5 ± 2.8^c^	48.5 ± 10.2^a, b, e^	32.0 ± 8.7^e^	7.2 ± 0.8^c, d^
Cortisol (µg/dl)	23.6 ± 2.0^b, c, d, e^	15.3 ± 2.3^a^	10.0 ± 1.1^a^	11.3 ± 1.3^a^	11.7 ± 0.3^a^

Significant difference (Scheffe’s multiple *t* test; *P* < 0.05): ^a^ *vs LEP*; ^b^ *vs LEPR*; ^c^ *vs MC4R;* ^d^ *vs* SvOb, ^e^ *vs* NWC.

The mean serum levels of OPN were comparable in individuals with *MC4R* deficiency and children with normal body weight and were significantly higher than those in the *LEP* and *LEPR* mutant groups (Table 2). The mean serum OPN levels observed in children with severe obesity with unknown genetic etiology were comparable to those in children with *LEP* or *LEPR* deficiency. No statistically significant difference was evident in the levels of the bone resorption biomarkers SOST and OPG in children with severe obesity compared to children with normal body weight.

### Endocrine profile

As expected, serum leptin levels were undetectable in children with *LEP* variants. Hyperleptinemia was evident in the remaining three groups of children with obesity and was most severe in children with *LEPR* deficiency (Table 2). The mean leptin levels in individuals with *MC4R* deficiency were comparable to those of children with severe obesity with unknown genetic etiology.

Serum insulin levels were significantly elevated in children with *MC4R* deficiency compared to individuals with pathogenic biallelic variants in *LEP* or *LEPR* genes, and children with severe obesity with unknown genetic etiology. The mean insulin levels in children with *LEP* and *LEPR* variants tended to be higher than the control values; however, this difference was not statistically significant (Table 2).

Similarly, mean serum insulin concentrations in age-matched individuals with severe obesity of unknown genetic etiology though discernibly higher than the *LEP*- and *LEPR*-deficient, were lower compared to those of children with *MC4R* deficiency (32.0 ± 8.7 *vs* 48.5 ± 10.2 ng/ml) but the difference was not statistically significant. Serum cortisol concentrations were significantly higher in children with *LEP* deficiency than in the other groups. No significant differences in serum cortisol levels were discernible in the other three groups of children with obesity and were comparable to those of the children with normal body weight (Table 2).

## Discussion

Our findings demonstrate that in children with biallelic *MC4R* variants, there is a 2–3-fold increase in the bone formation biomarkers, OC, and OPN, in comparison to the relatively lower levels of osteogenic markers observed in other children with obesity within our cohort. This contrasts with earlier studies that showed no significant changes in serum OC levels in children with only heterozygous *MC4R* variants [26]. This suggests that partial maintenance of MC4R integrity is sufficient to maintain normal osteogenesis. However, our results corroborate previous findings that used dual-energy X-ray absorptiometry scans to demonstrate increased bone mass in *MC4R* deficiency [31, 33]. Mice with *Mc4r* deficiency have also been shown to exhibit increased bone formation [26]. Additionally, increased body height has been reported in adolescents and adults with *MC4R* deficiency [5, 42]. Although the precise molecular events involved in increased bone formation as a result of MC4R dysfunction are yet to be elucidated, the main contributing factor to this increased bone mass is ascribed to CART overexpression. In turn, CART inhibits the osteoclastogenic factor receptor activator of nuclear factor kappa B ligand (RANKL), thereby decreasing bone resorption [43, 44].

In contrast, no conclusive data are available on bone resorption markers in early-onset severe obesity. A decrease in the levels of the bone resorption markers carboxy-terminal telopeptides of type-I collagen [26] and deoxypyridinoline [25] in individuals with pathogenic variants in *MC4R* has previously been reported. In our study, the levels of serum SOST, a biomarker for bone resorption [45], tended to be lower in the *MC4R* group than in the other groups. However, this trend needs to be confirmed in a larger cohort. This suggests that *MC4R* complete invalidation has a stronger effect on osteogenesis than on bone resorption. In contrast, in children with leptin-signaling deficiency due to loss-of-function biallelic variants in *LEP* or *LEPR*, reduced osteogenic activity was evidenced by significantly decreased serum levels of OC and OPN compared to the reference values in age-matched children with normal body weight. Importantly, in our group of children with severe consanguineous obesity of unknown genetic etiology, the profile of bone metabolic biomarkers was comparable to that of children with leptin-signaling deficiency. A deficit in bone formation in children with elevated leptin levels could partly be due to ‘leptin resistance’ or ‘cellular leptin resistance’ [46]. The possible mechanisms underlying leptin resistance associated with hyperleptinemia in rodents and humans have been extensively reviewed [46].

In individuals with general or monogenic forms of obesity, hyperleptinemia resulting from excessive fat mass can lead to hypothalamic and cellular leptin resistance, thereby disrupting the negative feedback of leptin on hypothalamic neurons (e.g., POMC, AgRP). *MC4R* deficiency has, therefore, been termed as the ‘purest’ form of leptin resistance [47]. Leptin also increases the expression of CART, which suppresses RANKL, thereby inhibiting bone resorption and favoring bone formation [25]. These factors disrupt the delicate balance of bone remodeling by promoting osteoclastogenesis and inhibiting osteoblast activity, ultimately favoring bone resorption over bone formation. Such processes also contribute to a paradoxical state in which, despite the higher bone mineral density often observed in individuals with obesity, their bones exhibit reduced quality and structural integrity, resulting in an increased risk of fractures [48–50]. The reduced osteogenic activity observed in cases of severe obesity could also be partly due to the absence of leptin’s regulatory effects on other hormones and factors, such as cytokines, which are crucial for normal bone growth and maintenance [17]. Our analysis did not reveal significant changes in the bone resorption biomarkers SOST and OPG in children with *LEP* or *LEPR* deficiency compared to those of children with normal weight (Table 2). According to a recent consensus, leptin has a stabilizing effect on bone metabolism and influences the simultaneous processes of bone formation and resorption. By binding to its receptors in the ventromedial nucleus of the hypothalamus, leptin increases adrenergic tone, which inhibits CART and activates RANKL, thereby promoting osteoclastogenesis. As leptin receptors have been identified in osteoblasts, leptin may also affect peripheral bone formation by the direct activation of these cells [25].

As anticipated, in our study participants, serum leptin levels were undetectable (<0.5 ng/ml) in *LEP* mutants and markedly elevated in children with *LEPR* and *MC4R* deficiencies. In addition, hyperleptinemia has previously been reported in patients with *LEPR* and *MC4R* deficiencies [51] and has been attributed to a lack of responsiveness to leptin signaling [52]. The mean serum insulin values were remarkably higher (*P* < 0.05) in children with *MC4R* deficiency than those in the other groups. Consistent with our observations, elevated insulin levels and hyperleptinemia have been reported in children with *MC4R* deficiency [31, 53]. We also observed significantly increased cortisol levels in children with *LEP* deficiency compared to the other three groups of patients with obesity, possibly due to a higher chronic stress level in children with *LEP* deficiency. These findings are consistent with those of a previous study reporting hypercortisolemia in children with *LEP* deficiency [51].

### Limitations

The main limitation of this study was the relatively small sample size, as biallelic monogenic variants leading to early-onset severe obesity are extremely rare world-wide, and the overall number of confirmed cases of monogenic obesity remains limited. The predominance of *LEP* mutation in our study highlights a distinct and relatively higher prevalence compared to other variants leading to monogenic obesity in the Pakistani population. Furthermore, the affected patients were to be selected within a narrow age range to achieve meaningful group comparisons. However, the late onset of obesity observed in MC4R subjects compared to those with *LEP* and *LEPR* variants, the mean age of presentation in children in this group remains slightly higher compared to the children with LEP or LEPR deficiency. The predominance of female participants in the LEP group reflects the random recruitment pattern at our center for this age group rather than a known sex-specific distribution of LEP mutations. In addition, the affected children were recruited from the outpatient departments of hospitals and, in most cases, had to travel from considerable distances. It was, therefore, not possible to draw fasting blood samples from these subjects at a fixed time. We also recognize that future investigations of bone turnover in patients with monogenic obesity should include the use of other specific bone metabolism markers, such as collagen peptide (PINP) and telopeptide (CTX). Additionally, we acknowledge that radiographic observations on the bone health of these patients could not be carried out in parallel due to logistical constraints. Finally, assessment of bone health using bone turnover markers could serve as a time and cost-effective, first-line investigation for suspected bone anomalies in children with obesity, before conducting more detailed confirmatory procedures, such as dual-energy X-ray absorptiometry.

## Conclusion

To our understanding, the present study is the first to investigate comparative bone metabolism in a series of children with genetically defined monogenic obesity from a single population, by using bone turnover biomarkers. Our findings suggest a potential trend towards increased osteogenic activity in children with biallelic MC4R deficiency. Conversely, a pronounced remission of bone formation was observed in children with leptin-signaling deficiency, supporting the osteogenic role of leptin. A similar tendency of bone remission was noted in children with severe obesity, negative for known genetic causes, possibly due to impaired leptin signaling/resistance. Our data support the view that severe obesity, particularly in children, does not necessarily lead to abnormal bone metabolism but is related to the etiology of the affliction. Based on present data, we believe that it is desirable to use specific bone turnover markers in addition to imaging techniques for a comprehensive evaluation of bone health in children with the onset of severe obesity at an early age, allowing for informed decisions about possible options for its management. Recombinant leptin has been used to treat children with leptin deficiency for more than two decades and has been reported to have a beneficial effect on bone health [54]. More recently, setmelanotide, an MC4R agonist that bypasses leptin resistance, has been approved for the treatment of monogenic obesity due to *LEPR*-, *POMC*-, or *PCSK1*-deficient states [55–57]. However, the effect of this agonist on bone health in these forms of monogenic obesity remains to be elucidated on a long-term basis. The advent of new drugs based on GLP and GIP receptor agonists [58, 59] for the treatment of common obesity necessitates a comprehensive evaluation of bone health and metabolism. In initial studies, GLP-1 receptor agonists and their analogs have recently been investigated in preclinical trials to improve bone fragility disorders [59].

## Data Availability

The individual data supporting the findings of this study are not publicly available as they are the property of the University of Lille, France, and the Forman Christian College (A Chartered University), Lahore, Pakistan. Participants in the study did not consent to share their data outside of publication. All data generated or analyzed in this study are included in this article. To access the data, please contact the corresponding authors—Philippe Froguel (p.froguel@imperial.ac.uk) or Muhammad Arslan (muhammadarslan@fccollege.edu.pk).
